# Intracardiac ECG for confirmation of correct positioning of central venous catheters is safe and cost-effective

**DOI:** 10.1186/cc12113

**Published:** 2013-03-19

**Authors:** M Eriksson, R Dörenberg

**Affiliations:** 1 Surgical Sciences, Uppsala, Sweden

## Introduction

About 10 years ago the use of chest radiographs as the golden standard to ensure correct positioning of central venous catheters (CVC) was questioned. The frequent use of CVCs was also challenged. We decided to retrospectively evaluate our routines in a large surgical unit in a Swedish university hospital.

## Methods

All X-rays were centrally registered. Chest X-ray performed in our unit is almost entirely used to confirm CVC positioning. The Certofix CVC set for the Seldinger technique in combination with Certodyn - Universaladapter (B Braun, Germany) is now used as the routine equipment and the right jugular vein is our standard approach.

## Results

In 2002 the total number of X-rays performed in patients at our unit was 2,306, which corresponds to the approximate number of inserted CVCs at that time, since a confirmatory X-ray was routine. X-rays were rarely performed on other indications in our unit. X-ray costs were at that time approximately €300,000 (~€130/each). The year after, 1,726 chest X-rays were performed, reflecting both the use of intracardiac confirmation of correct CVC position and also a reduced use of CVCs. This trend has continued over time. In 2011 approximately 600 CVCs were inserted at our unit. X-rays were performed in about 20% of these cases. The cost for a chest X-ray is today ~€200, meaning that X-ray costs were approximately €24,000. We have not experienced any medical problems when intracardiac ECG was used for positioning confirmation. On the contrary, aspiration of venous blood without apparent p-waves in a patient with sinus rhythm may suggest improper placement of the CVC; for example, the right brachial vein.

## Conclusion

If we had continued to use CVCs at the same frequency as we did 10 years ago, and used X-ray confirmation in practically all cases, we would have paid approximately €460,000 annually. Reduced use of CVCs, in combination with intracardiac confirmation of CVC positioning, has not only allowed us to reduce costs associated with CVC insertion by more than €400,000, corresponding to a reduction rate of more than 90%, but also decreased the patient's exposure to X-ray irradiation.
